# Supplementary material for: Nascent liver proteome reveals enzymes and transcription regulators under physiological and alcohol exposure conditions
Source: Nat Commun. 2025 Aug 26;16:7945. doi: 10.1038/s41467-025-63212-9 (PMC12381119; doi:10.1038/s41467-025-63212-9)
Supplement: Supplementary file 1 — Supplementary Information [file 41467_2025_63212_MOESM1_ESM.pdf]

Supplementary Information for:

Nascent liver proteome reveals enzymes and transcription regulators under physiological and alcohol exposure conditions

Jiayu Gu[1,†], Lihui Lao[2,†], Linzhen Hu[2], Jia Zang[2], Chao Liu[2,3], Ruixi Wan[2], Ling Tang[2], Ying Yuan[1*], Yulin Chen[1,2,3*], Shixian Lin[1,2,3,4,5*]

[1]Department of Medical Oncology, Laboratory of Cancer Prevention and Intervention Ministry of Education, The Second Affiliated Hospital, Zhejiang University School of Medicine, Hangzhou, China. [2]Zhejiang Provincial Key Laboratory for Cancer Molecular Cell Biology, Life Sciences Institute, Zhejiang University, Hangzhou, China. [3]Shaoxing Institute, Zhejiang University, Shaoxing, China. [4]Institute of Fundamental and Transdisciplinary Research, Zhejiang University, Hangzhou, China. [5]State Key Laboratory of Transvascular Implantation Devices, The Second Affiliated Hospital, Zhejiang University School of Medicine, Hangzhou, China. [†]These authors contributed equally to this work. *Correspondence: sxlin@zju.edu.cn; chenyulin@zju.edu.cn; yuanying1999@zju.edu.cn

- **Supplementary Figures**

Supplementary Figure 1. PylRS variants screening and SORT labeling in HEK293T.

Supplementary Figure 2. Construction and characterization of SORT$_{KASM}$ mouse model.

Supplementary Figure 3. Optimization of click chemistry reaction conditions of nascent proteome in the mouse liver.

Supplementary Figure 4. Analysis of nascent proteome in the mouse liver under physiological condition.

Supplementary Figure 5. Characterization and enrichment of the nascent liver proteome of ethanol-induced liver injury mouse model.

Supplementary Figure 6. Analysis of the nascent liver proteome in an ethanol-induced liver injury mouse model.

Supplementary Figure 7. Validating the function of Hsp and Acsl1/5 involved in alcohol-induced lipid accumulation.

Supplementary Figures

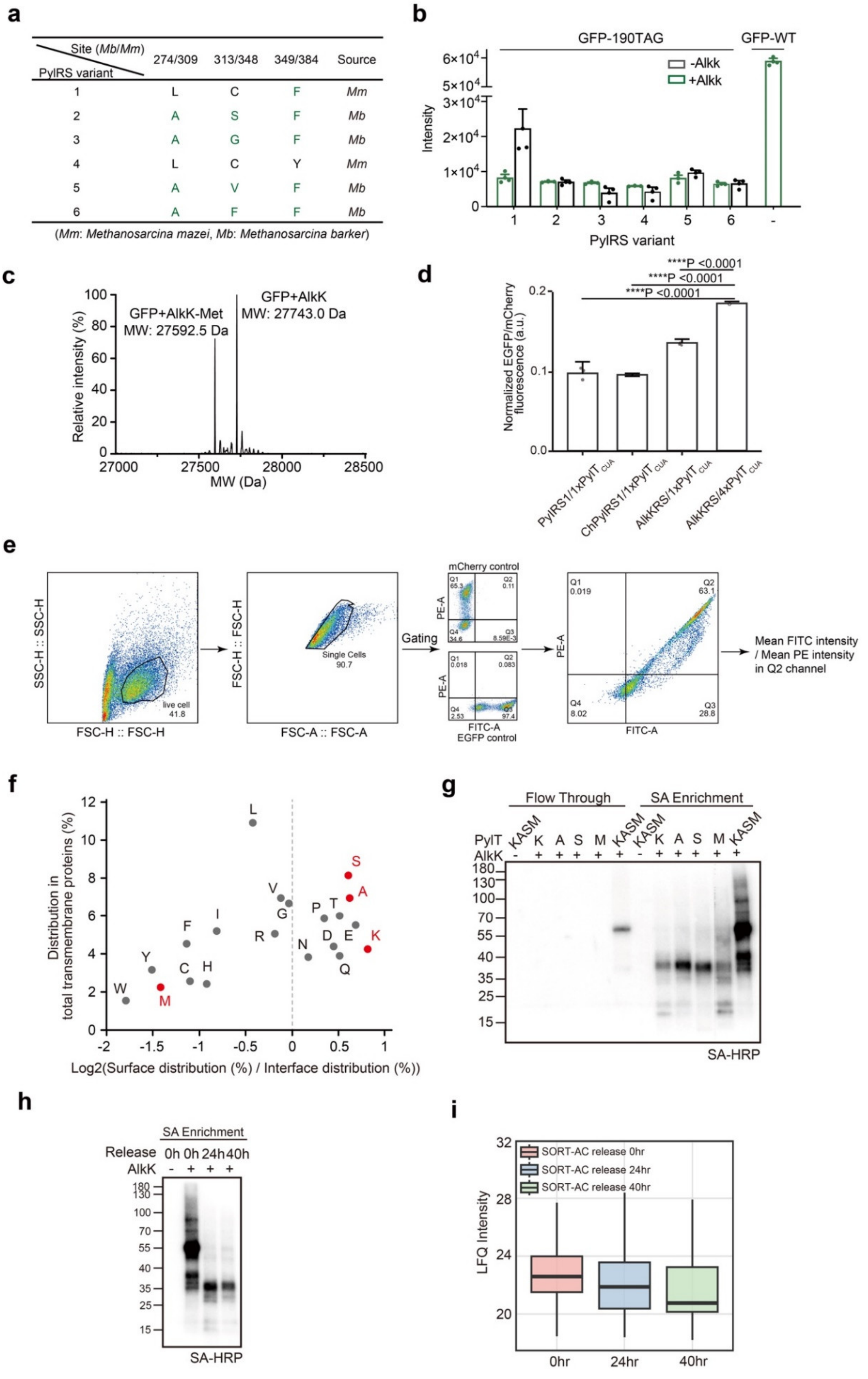

Supplementary Figure 1. PylRS variants screening and SORT labeling in HEK293T. a, PylRS variants and the respective mutation sites. The mutated amino acids were colored in green. *Mm*, *Methanosarcina mazei*. *Mb*, *methanosarcina barkeri*. **b,** GFP reporter assay for amber suppression efficiency. The fluorescent intensity in each group is measured by a plate reader and normalized by the GFP-WT. Data are the mean ± s.d.; n = 3 biologically independent repeats. **c,** Mass spectrometry characterizes the fidelity of AlkK incorporation into GFP reporter. **d,** Quantification analysis of amber suppression activity of the different PylRS variants by GFP reporter assay using FACS of **Fig. 1e**. Statistical significance was determined using one-way ANOVA with Tukey's multiple comparison test. Data are the mean ± s.d.; n = 3 biologically independent repeats. **e,** The gate strategy of FACS used in **Fig.1e**. Live, single cells were gated based on FSC/SSC and FSC-A versus FSC-H, with fluorescence quadrants defined using single-color controls. Amber suppression efficiency was quantified as the ratio of FITC to PE MFI in $EGFP^{+}mCherry^{+}$ (Q2) cells. **f,** Analysis of 20 natural amino acids distribution on human transmembrane proteins, and the ratio of 20 natural amino acids distribution on surface and interface on proteins. The residues selected for the following randomly incorporation of AlkK were colored in red. **g,** Nascent proteomes labeling after click reaction and streptavidin resin enrichment in HEK293T using K, A, S, M individually and in combination. Experiment was independently repeated three times. **h,** Nascent proteomes enrichment at different time points (0 h to 40 h) after AlkK withdrawal in HEK293T. Experiment was independently repeated three times. **i,** Box plot showing the distribution of LFQ intensity values of nascent proteins identified by SORT-AC at 0 h, 24 h, and 40 h after AlkK withdrawal. Source data are provided as a Source Data file.

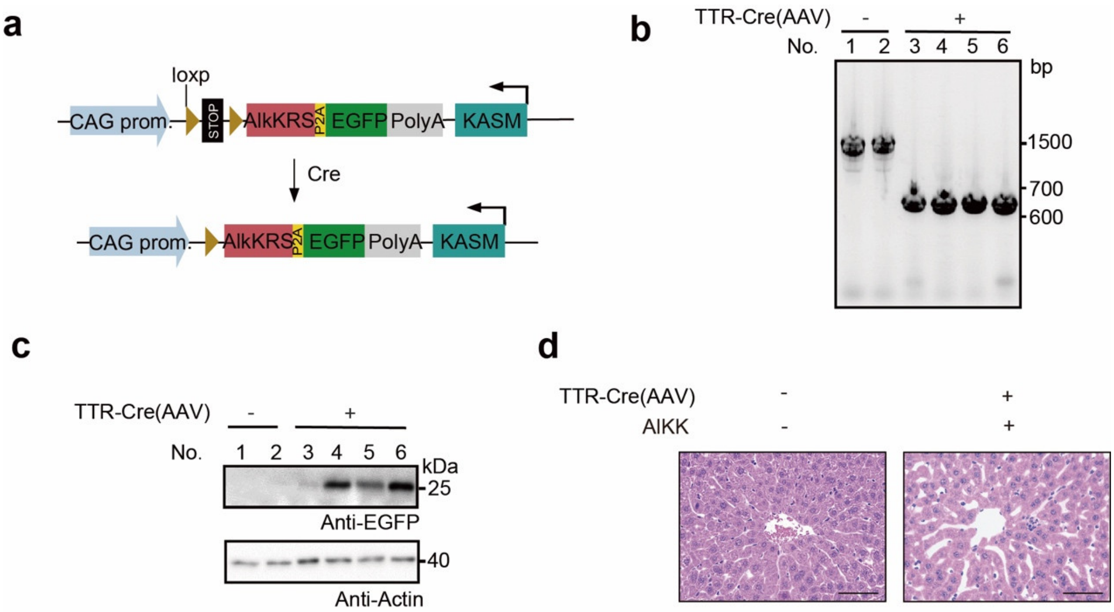

Supplementary Figure 2. Construction and characterization of SORT$_{KASM}$ mouse model. a, The strategy for constructing genetically conditional knock-in mice SORT$_{KASM}$. Using CRISPR/Cas9 technology, the Cas9 protein binds to the H11 locus guided by gRNA, causing DNA double-strand breaks. The donor vector repairs the broken double strands through homologous recombination, thus achieving gene knock-in at the target site. **b,** Cre induced FloxP-STOP excision detected by genotyping. Mice 1 and 2, injected with PBS in the orbital area, did not show the FloxP-STOP excised, while mice 3, 4, 5, and 6, injected with TTR-Cre-AAV in the orbital area, had the FloxP-STOP excised. **c,** Immunoblotting of EGFP signals (AlkKRS) detected in the liver tissues of mice that received injections of AAV carried TTR-Cre and supplemented with AlkK. Experiment was independently repeated three times. **d,** Comparison of liver tissue sections stained with HE between mice injected with AAV and drinking AlkK and normal mice. Scale bar, 100μm. Representative images of n = 3 mice. Source data are provided as a Source Data file.

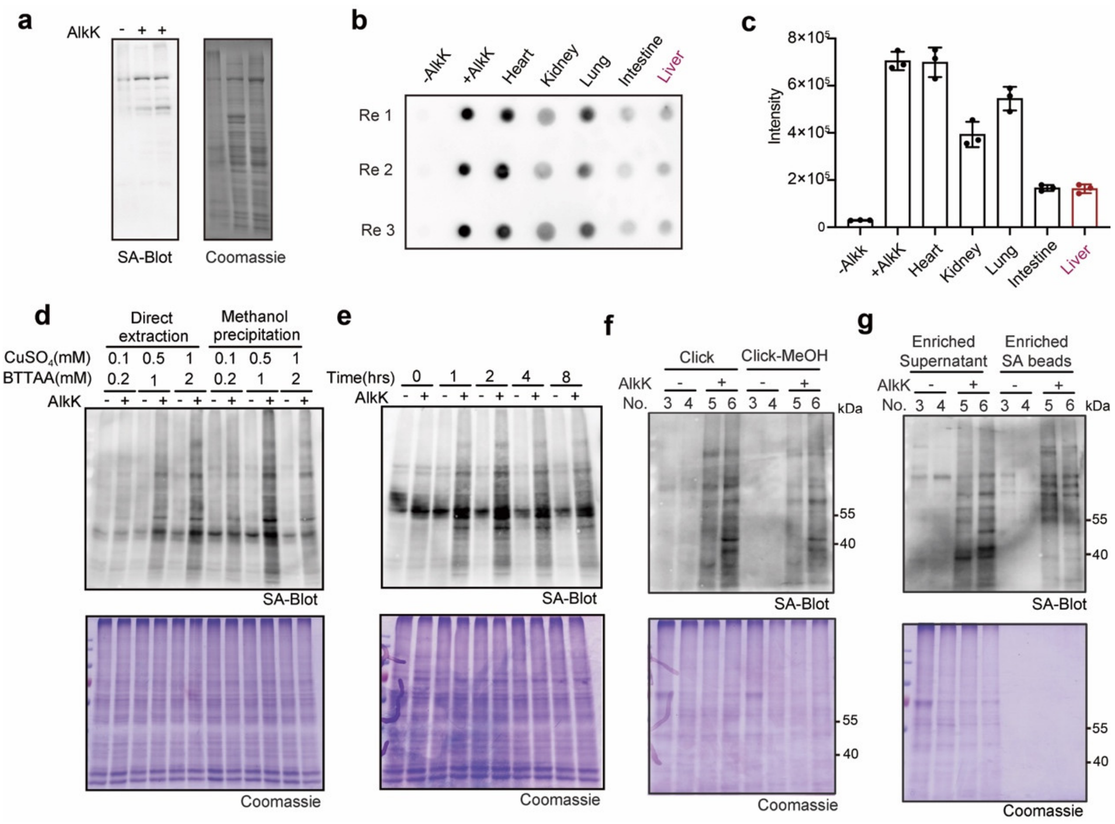

Supplementary Figure 3. Optimization of click chemistry reaction conditions of nascent proteome in the mouse liver. a, Labeling under pre-optimized click reaction conditions of liver tissue in $SORT_{KASM}$ mice. SA-Blot, immunoblotting with streptavidin-HRP. **b-c,** Cells transfected with the SORT system and cultured in the presence of AlkK were mixed with tissue samples from mouse liver, heart, kidney, and other organs, followed by a click reaction. The quantified of streptavidin signal intensities are shown in **c**. Data are the mean ± s.d.; n = 3 biologically independent repeats. **d-f,** Optimization of the conditions for the azide-alkyne cycloaddition reaction on the concentration of Cu^{2+} and the BTTAA ligand (**d**), the method of protein extraction (**d**) and reaction time (**e**). The original labeling result before condition optimization was show at the lane 1 and 2 in (**d**). **f-g,** Labeling(**f**) and enrichment(**g**) of the nascent proteins of the whole proteome by western blot. Full blot of Fig. 3b. SA-Blot, immunoblotting with streptavidin-HRP. Experiment was independently repeated three times. Source data are provided as a Source Data file.

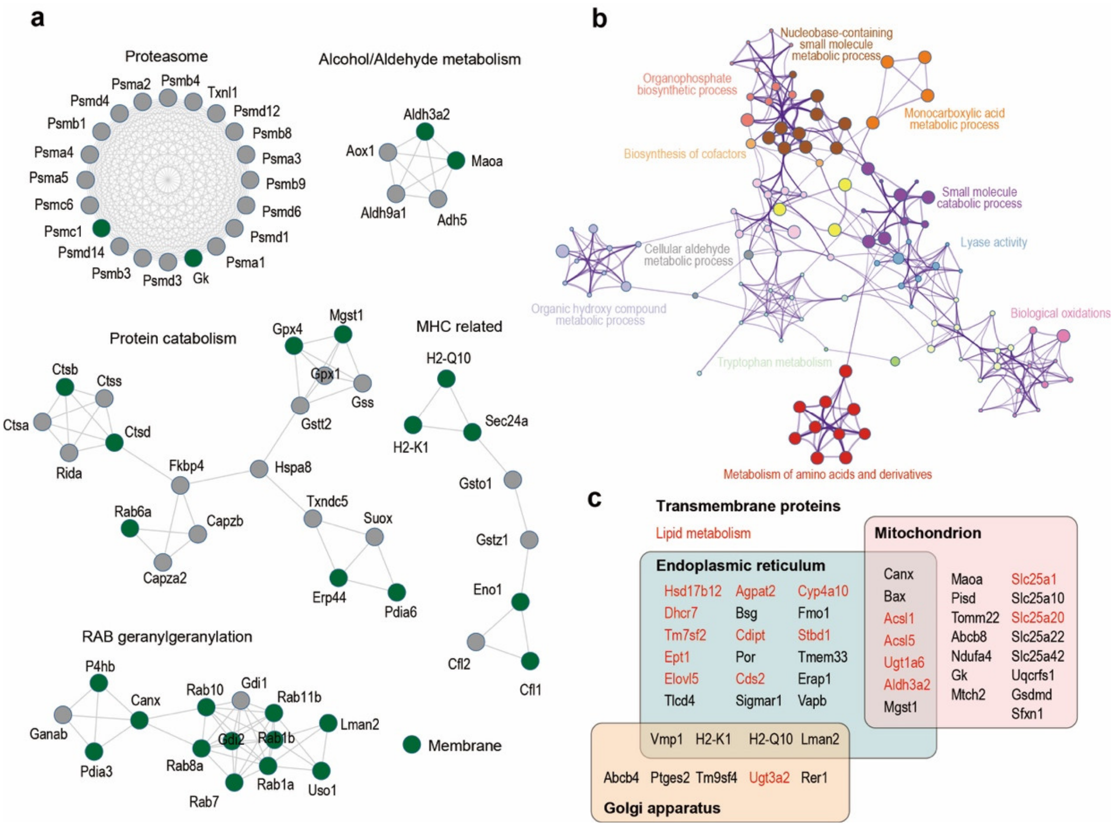

Supplementary Figure 4. Analysis of nascent proteome in the mouse liver under physiological condition. a, The interaction network analysis of SORT-AC Print proteins. Full network of Fig. 3h. **b,** The pathway network analysis of SORT-AC Print proteins. **c,** Overlap of transmembrane proteins in SORT-AC Print in mitochondrion, endoplasmic reticulum and Golgi apparatus. Proteins involved in lipid metabolism were colored in red.

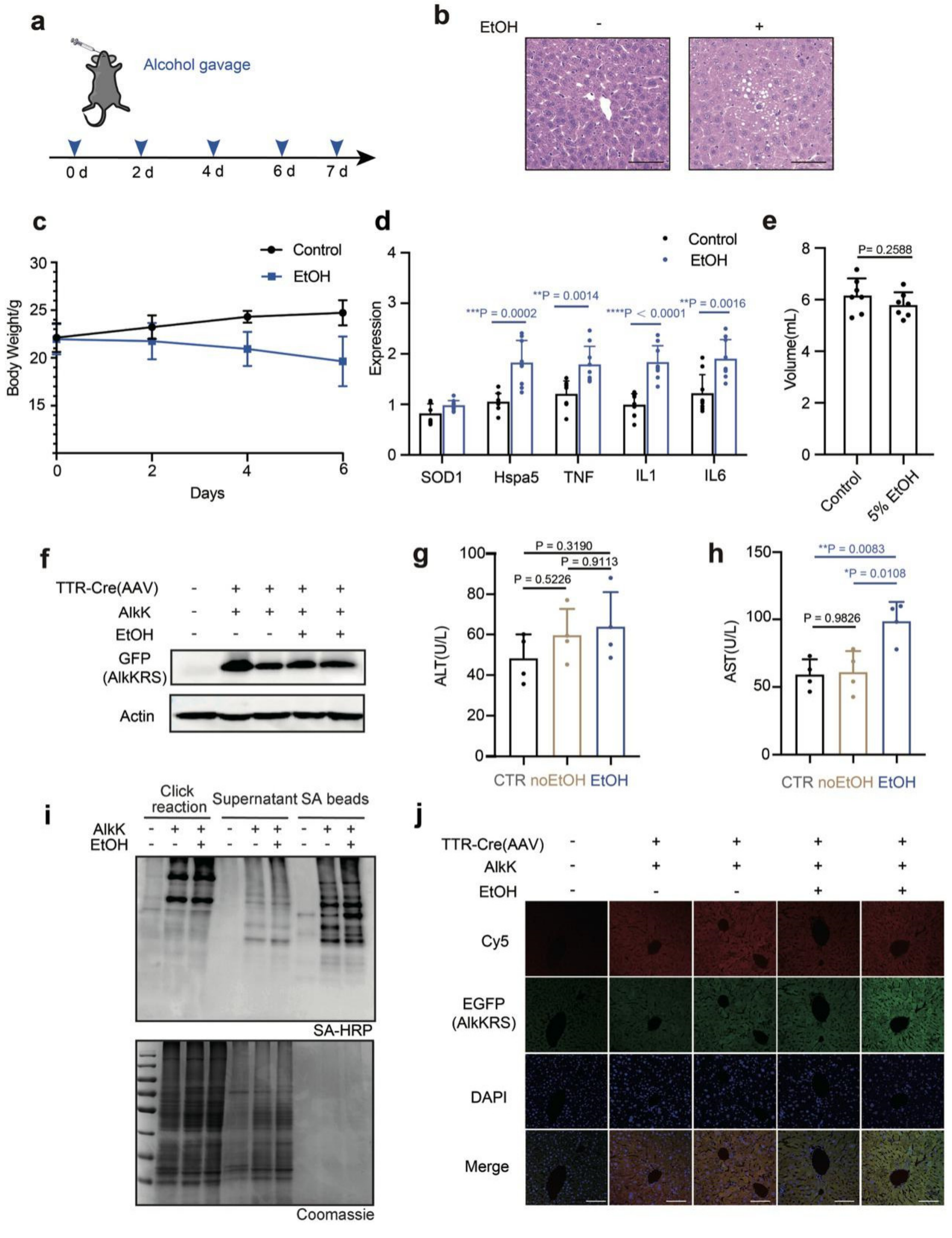

Supplementary Figure 5. Characterization and Enrichment of the nascent liver proteome of ethanol-induced liver injury mouse model. a-d, Pilot study of the mouse alcohol exposure model. Mice were subjected to an intermittent ethanol gavage protocol (4 g/kg, every other day for 4 doses), with an additional gavage administered one day prior to sample collection (**a**). Representative HE staining of liver tissue

revealed steatosis (**b**). A moderate reduction in body weight was observed during the gavage period (**c**) and qPCR analysis confirmed the upregulation of inflammation-related genes in the hepatic transcriptome (**d**). Scale bar, 100μm. Data are the mean ± s.d.; n = 5 mice. **e,** The effect of 5% ethanol on water intake in mice. Two groups of mice were administered either water or water containing 5% ethanol. The average daily fluid intake was monitored over a 7-day period. No significant difference was observed between control and ethanol-treated group. Data are the mean ± s.d.; n = 3 mice. **f,** Immunoblotting of EGFP signals (AlkKRS) detected in the liver tissues of mice that received injections of AAV carried TTR-Cre and supplemented with AlkK. Experiment was independently repeated three times. **g-h,** The elevated levels of serum AST and ALT in mice indicate the effectiveness of the ethanol-induced liver injury mouse model. Statistical significance was determined using one-way ANOVA with Tukey's multiple comparison test. Data are the mean ± s.d.; n = 4 mice. **i,** Labeling and enrichment of the nascent proteins in liver tissue by SORT-AC in mouse with or without AlkK and alcohol treatment. Experiment was independently repeated three times. **j,** The fluorescence scanning of SORT-AC in ethanol-induced liver injury mouse model liver sections. EGFP signal represented the AlkKRS expression level in liver tissue. Cy5 signal represented the labeled nascent proteome. Representative images of n = 3 experiments. Scale bar, 100μm. Source data are provided as a Source Data file.

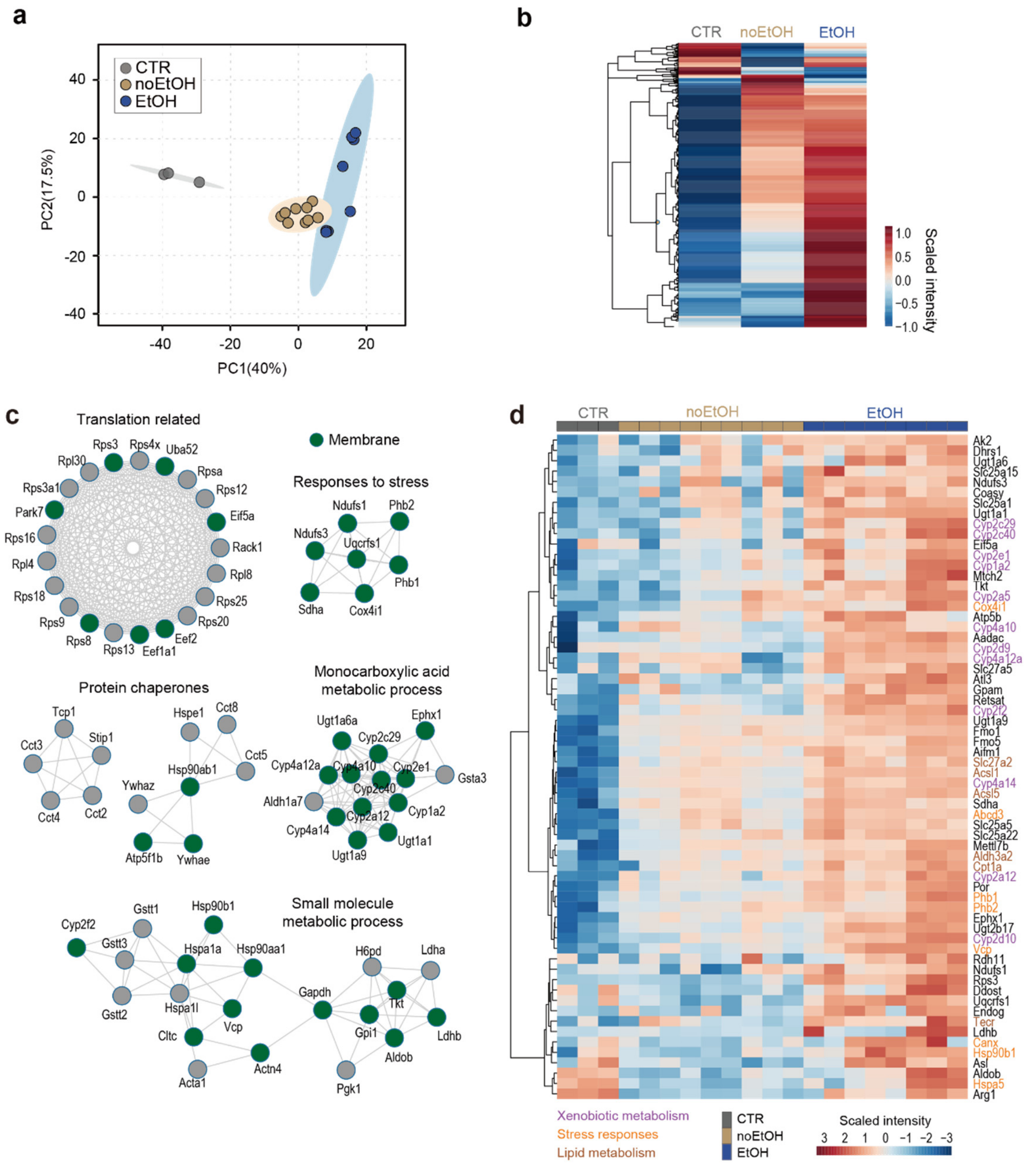

Supplementary Figure 6. Analysis of the nascent liver proteome in an ethanol-induced liver injury mouse model. a, Principal component analysis (PCA) showed an excellent biological reproducibility between repeats. **b,** LFQ analysis with heatmap showed a dramatic increase of newly synthesized protein level and number in EtOH group. **c,** The interaction network analysis of EtOH Enrich proteins. **d,** The heatmap analysis of EtOH Enriched proteins on the membrane of ER or mitochondrion. The proteins participated in xenobiotic metabolism is colored in purple, in stress responses in colored in yellow, in lipid metabolism in colored in brown.

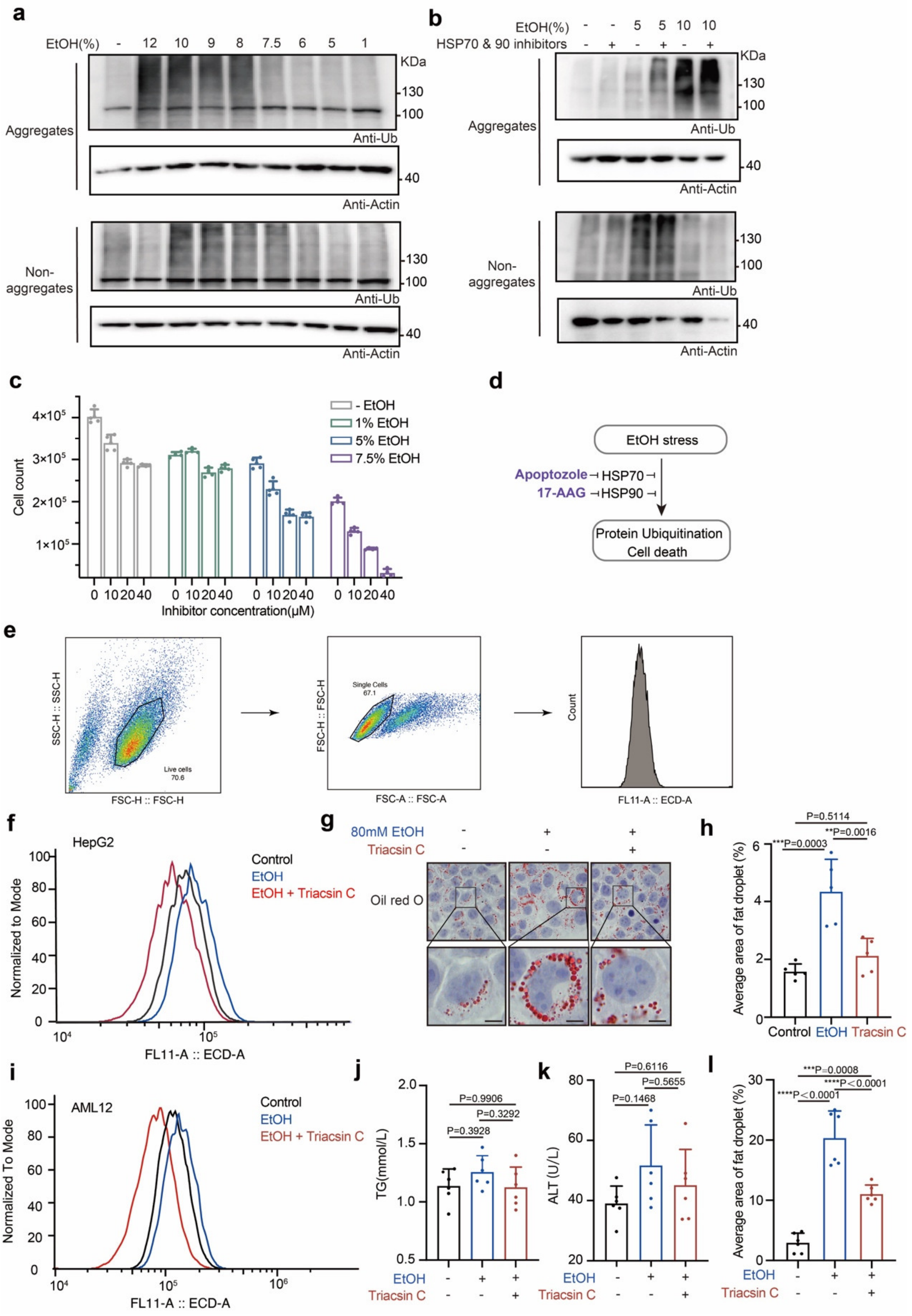

Supplementary Figure 7. Validating the function of Hsp and Acsl1/5 involved in alcohol-induced lipid accumulation. a, The ubiquitination level of aggregates and

non-aggregate fractions in HepG2 cells treated with different ethanol concentrations using western blot. **b,** Hsp70 and Hsp90 inhibitors (Apoptozole and 17-AAG) could promote the ubiquitination of protein under different ethanol concentrations, while it did not show accumulation of ubiquitination in non-aggregate fractions. **c,** Survival analysis of the HepG2 cells exposed in different ethanol concentration treated with different concentrations of Hsp70 and Hsp90 inhibitors. Data are the mean ± s.d.; n = 5 biologically independent repeats. **d,** The schematic of the function of Hsp proteins and inhibitors in protein aggregation and cell death. **e,** The gate strategy of FACS used in **f** and **i**. Cells were stained with Nile Red to label intracellular lipid droplets. Live cells were first gated based on FSC and SSC profiles. Single cells were selected by FSC-A vs. FSC-H to exclude doublets. The fluorescence intensity histogram of ECD-A channel displays the Nile Red staining intensity of the cells. **f**, In HepG2 cells， Triacsin C could effectively decrease lipid accumulation compared with the same ethanol treatment without Triacsin C using FACS through Nile Red staining. **g,** Oil Red O staining of AML12 cells treated with or without Triacsin C. Representative images of n = 3 experiments. **h,** Quantification analysis of **g**. Statistical significance and P values were determined using one-way ANOVA with Tukey's multiple comparison test. Data are the mean ± s.d.; n = 5. **i,** Validation of the inhibitory effect of Triacsin C on ethanol-induced lipid accumulation in AML12 cells. **j-k**, Comparison of serum ALT and TG levels between alcohol-induced liver injury mouse models treated and untreated with Triacsin C. Statistical significance and P values were calculated by one-way ANOVA with Tukey's multiple comparison test. Data are the mean ± s.d.; n = 6 mice. **l,** Statistical analysis of Oil Red O staining in liver sections from mice treated with or without treatment of Triacsin C. Statistical significance was determined using one-way ANOVA with Tukey's multiple comparison test. Data are the mean ± s.d.; n = 6 mice. Source data are provided as a Source Data file.
