# Supplementary material for: Nascent liver proteome reveals enzymes and transcription regulators under physiological and alcohol exposure conditions
Source: Nat Commun. 2025 Aug 26;16:7945. doi: 10.1038/s41467-025-63212-9 (PMC12381119; doi:10.1038/s41467-025-63212-9)
Supplement: Supplementary file 2 — Description of Additional Supplementary Files [file 41467_2025_63212_MOESM2_ESM.pdf]

Description of Additional Supplementary Files

File Name: Supplementary Data 1. SORT-labeled proteome in HEK293T.

Description: This table contains stepwise analysis of nascent proteomes in HEK293T cells transfected with PylT-K, -A, -S, or -M individually or in combination. Time-course profiling of nascent proteomes labeled in PylT-KASM-transfected cells harvested at 0 to 40 h following AlkK withdrawal is also included. Membrane protein annotation was performed by mapping identified proteins to the UniProtKB database.

File Name: Supplementary Data 2. SORT-AC in liver of mouse at physiological state.

Description: The table lists the quantification analysis of nascent proteomes labeled in mouse liver at physiological state. Furthermore, it includes the liver-specific expression data derived from the STRING database, as well as membrane protein identification based on UniProtKB annotations. The detailed analysis of membrane protein localization by UniProt annotation is also provided.

File Name: Supplementary Data 3. SORT-AC in liver of ethanol-induced liver injury mouse model.

Description: The table summarizes the quantification analysis of nascent proteomes labeled in mouse liver under alcoholic conditions. It further includes membrane protein identification through mapping to the UniProtKB database, accompanied by detailed subcellular localization information based on UniProt annotations.

File Name: Supplementary Data 4. Sequences of oligonucleotides.

Description: The table presents the guide RNA sequences and corresponding PAM sequences used for the generation of the $SORT_{KASM}$ mouse line, along with the genotyping primers specific to this model. Additionally, it includes the full list of primer sequences utilized for qPCR analyses in this study.
