# Supplementary material for: Nascent liver proteome reveals enzymes and transcription regulators under physiological and alcohol exposure conditions
Source: Nat Commun. 2025 Aug 26;16:7945. doi: 10.1038/s41467-025-63212-9 (PMC12381119; doi:10.1038/s41467-025-63212-9)
Supplement: Supplementary file 7 — Reporting Summary [file 41467_2025_63212_MOESM7_ESM.pdf]

nature portfolio

Corresponding author(s): Shixian Lin, Yulin Chen, Ying Yuan

Last updated by author(s): Jun 5, 2025

Reporting Summary

Nature Portfolio wishes to improve the reproducibility of the work that we publish. This form provides structure for consistency and transparency in reporting. For further information on Nature Portfolio policies, see our Editorial Policies and the Editorial Policy Checklist.

Statistics

For all statistical analyses, confirm that the following items are present in the figure legend, table legend, main text, or Methods section.

n/a	Confirmed	
☐	☒	The exact sample size (n) for each experimental group/condition, given as a discrete number and unit of measurement
☐	☒	A statement on whether measurements were taken from distinct samples or whether the same sample was measured repeatedly
☐	☒	The statistical test(s) used AND whether they are one- or two-sided<br>*Only common tests should be described solely by name; describe more complex techniques in the Methods section.*
☒	☐	A description of all covariates tested
☒	☐	A description of any assumptions or corrections, such as tests of normality and adjustment for multiple comparisons
☐	☒	A full description of the statistical parameters including central tendency (e.g. means) or other basic estimates (e.g. regression coefficient) AND variation (e.g. standard deviation) or associated estimates of uncertainty (e.g. confidence intervals)
☐	☒	For null hypothesis testing, the test statistic (e.g. F, t, r) with confidence intervals, effect sizes, degrees of freedom and P value noted<br>*Give P values as exact values whenever suitable.*
☒	☐	For Bayesian analysis, information on the choice of priors and Markov chain Monte Carlo settings
☒	☐	For hierarchical and complex designs, identification of the appropriate level for tests and full reporting of outcomes
☒	☐	Estimates of effect sizes (e.g. Cohen's d, Pearson's r), indicating how they were calculated

Our web collection on statistics for biologists contains articles on many of the points above.

Software and code

Policy information about availability of computer code

Data collection	Optical density at 600 nm wavelength (OD600) and fluorescence intensity were acquired with a BioTek Synergy Neo2 multifunctional microplate reader with Gen5 CHS 2.09 software. Chemilumine scence of western blot was captured by Azure Biosystems C400 with cSeries Capture Software (version 2.1.4.731, Azure Biosystems). FACS data were collected on a Beckman CytoFlex with CytExpert (version 2.0.0.153, Beckman Coulter). LC-MS analysis was performed on a Xevo G2-XS QTOF MS system (Waters Corporation) with UNIFI software (version 1.9.4, Waters). LC-MS/MS raw files were acquired by Thermo Scientific Q Exactive HF-X Orbitrap and Orbitrap Exploris 480 mass spectrometers with Xcalibur (version 3.0.63, Thermo Fisher Scientific Inc.). Fluorescence images were collected using ZEISS LSM 880 and LSM 900 Confocal microscope with ZEN software. The qPCR results were acquired by Bio-Rad CFX96 Connect. The serum was analysed for AST via Biochemistry Analyzers (Roche, COBASC 311). The oil red O staining was photographed under a BX53 microscope with DP25 camera (Olympus).
Data analysis	The assessment of amber suppression efficiency was processed with Origin 2017pro software (version 9.4). Deconvolution of LC-MS spectra was performed using UNIFI software (version 1.9.4, Waters). FACS data were processed with FlowJo V10 (version 14.0.0.0, Flexera Software). LC-MS/MS raw files were analyzed with MaxQuant (version 2.2.0.0), Perseus (version 2.0.7.0), Metascape web tool (version 3.5), Cytoscape (version 3.8.2), MetaboAnalyst web tool (version 6.0), R (version 4.2.0) and ggplot2 (version 3.4.2). Chemilumine scence was processed by ImageJ (version 1.52). Fluorescence and other microscopic images were analyzed by ZEN lite (version 2.3) and ImageJ (version 1.52). Statistical analysis and data presentation were performed using GraphPad Prism 9 and R (4.2.0). All figures and every element of these figures were created in the Adobe Illustrator (Version 2020).

For manuscripts utilizing custom algorithms or software that are central to the research but not yet described in published literature, software must be made available to editors and reviewers. We strongly encourage code deposition in a community repository (e.g. GitHub). See the Nature Portfolio guidelines for submitting code & software for further information.

Data

Policy information about availability of data

All manuscripts must include a data availability statement. This statement should provide the following information, where applicable:

- Accession codes, unique identifiers, or web links for publicly available datasets
- A description of any restrictions on data availability
- For clinical datasets or third party data, please ensure that the statement adheres to our policy

The mass spectrometry proteomics data have been deposited to the ProteomeXchange Consortium (http://proteomecentral.proteomexchange.org) via the iProX partner repository with the dataset identifier PXD051725. Previously published datasets that were used for reanalysis of the tissue specific. Swiss-Prot human database (Release 2025-04-16) containing 20,421 reviewed entries and Swiss-Prot mouse database (Release 2023-02-06) containing 17,145 reviewed entries from UniProt was used for proteomics analysis. Source data are provided with this study. All other data supporting the findings of this study are available from the corresponding author on reasonable request.

Research involving human participants, their data, or biological material

Policy information about studies with human participants or human data. See also policy information about sex, gender (identity/presentation), and sexual orientation and race, ethnicity and racism.

Reporting on sex and gender	No human research participants.
Reporting on race, ethnicity, or other socially relevant groupings	No human research participants.
Population characteristics	No human research participants.
Recruitment	No human research participants.
Ethics oversight	No human research participants.

Note that full information on the approval of the study protocol must also be provided in the manuscript.

Field-specific reporting

Please select the one below that is the best fit for your research. If you are not sure, read the appropriate sections before making your selection.

☒ Life sciences ☐ Behavioural & social sciences ☐ Ecological, evolutionary & environmental sciences

For a reference copy of the document with all sections, see nature.com/documents/nr-reporting-summary-flat.pdf

Life sciences study design

All studies must disclose on these points even when the disclosure is negative.

Sample size	No statistical method was used to pre-determine sample size, which was determined in accordance with standard practices in the field. Sample size for each experiment is indicated in the figure legend.
Data exclusions	No data were excluded from the analyses.
Replication	All experiment was repeated independently at least twice with similar results.
Randomization	Mammalian cells and mice used in this study were randomly assigned as controls or treatment groups. For proteomics, samples were analyzed in a randomized run order. The other experiments were not randomized as the order of analysis does not influence the experimental outcomes.
Blinding	The investigators were not blinded to allocation during experiments and outcome assessment. Data collection and analysis were not performed blind to the conditions of the experiments as the method of quantification with multiple replicates ensures unbiased processing.

Reporting for specific materials, systems and methods

We require information from authors about some types of materials, experimental systems and methods used in many studies. Here, indicate whether each material, system or method listed is relevant to your study. If you are not sure if a list item applies to your research, read the appropriate section before selecting a response.

Materials & experimental systems

n/a	Involved in the study	
☐	☒	Antibodies
☐	☒	Eukaryotic cell lines
☒	☐	Palaeontology and archaeology
☐	☒	Animals and other organisms
☒	☐	Clinical data
☒	☐	Dual use research of concern
☒	☐	Plants

Methods

n/a	Involved in the study	
☒	☐	ChIP-seq
☐	☒	Flow cytometry
☒	☐	MRI-based neuroimaging

Antibodies

Antibodies used	Primary antibodies:<br>Rabbit polyclonal anti-GFP (2555, Cell Signaling Technology, 1:1000),<br>Rabbit monoclonal anti-Actin, lot 314577 (Abmart, T40001, 1:1000),<br>Mouse polyclonal anti-His (Cell Signaling Technology, 2365, 1:1000),<br>Rabbit polyclonal anti-Ubiquitin (10201-2-AP, Proteintech, 1:1000),<br>Streptavidin-HRP (Cell Signaling Technology, 3999, 1:1000).<br><br>Secondary antibodies:<br>Anti-Mouse IgG, HRP-conjugated antibody (Proteintech, SA00001-1, 1:5000),<br>Anti-Rabbit IgG, HRP-conjugated antibody (Abmart, M21002, 1:5000),
Validation	All commercial antibodies used in this study can be found in the manufacturer's website for each product:<br>Rabbit polyclonal anti-GFP (2555, Cell Signaling Technology, 1:1000) was validated for WB and IHC, and relevant citation can be found on the manufacturer's website (https://www.cellsignal.com/products/primary-antibodies/gfp-antibody/2555).<br>Rabbit monoclonal anti-Actin, lot 314577 (Abmart, T40001, 1:1000) was validated for WB, and relevant citation can be found on the manufacturer's website (http://www.ab-mart.com.cn/page.aspx?node=%2059%20&id=%201021).<br>Mouse polyclonal anti-His (Cell Signaling Technology, 2365, 1:1000) was validated for WB and IP, and relevant citation can be found on the manufacturer's website (https://www.cellsignal.com/products/primary-antibodies/his-tag-antibody/2365).<br>Rabbit polyclonal anti-Ubiquitin (10201-2-AP, Proteintech, 1:1000) was validated for WB, IF and IHC, and relevant citation can be found on the manufacturer's website (https://www.ptglab.com/products/ubiquitin-Antibody-10201-2-AP.htm).<br>Streptavidin-HRP (Cell Signaling Technology, 3999, 1:1000) was validated for WB, and relevant citation can be found on the manufacturer's website (https://www.cellsignal.com/products/wb-ip-reagents/streptavidin-hrp/3999).<br>Anti-Mouse IgG, HRP-conjugated antibody (Proteintech, SA00001-1, 1:5000) was validated for WB, and relevant citation can be found on the manufacturer's website (https://www.ptglab.com/products/HRP-conjugated-Affinipure-Goat-Anti-Mouse-IgG-H-L-secondary-antibody.htm).<br>Anti-Rabbit IgG, HRP-conjugated antibody (Abmart, M21002, 1:5000) was validated for WB, and relevant citation can be found on the manufacturer's website (http://www.ab-mart.com.cn/page.aspx?node=%2062%20&id=%20980).

Eukaryotic cell lines

Policy information about cell lines and Sex and Gender in Research

Cell line source(s)	HEK293T, HepG2 and AML12 cell lines are from ATCC.
Authentication	None of the cell line used was authentication.
Mycoplasma contamination	Cells were confirmed to be free of mycoplasma contamination.
Commonly misidentified lines (See ICLAC register)	No commonly misidentified cell lines were used.

Animals and other research organisms

Policy information about studies involving animals; ARRIVE guidelines recommended for reporting animal research, and Sex and Gender in Research

Laboratory animals	The gene conditional knock-in mouse strains discussed in this article were constructed from C57BL/6J strain by Gempharmatech Co., Ltd. Utilizing CRISPR/Cas9 technology, SORT-AlkK-KASM system was insertion at H11 site was achieved through homologous recombination. Meanwhile, a loxP flanking stop sequence leading AlkKRS-P2A-EGFP is expressed from the CAG promoter, and PylTKASM is expressed from the U6 promoter. Parental heterozygous mice SORT-AlkK-KASM+/- are bred at a female-to-male ratio of 1:2 or 1:3. After the offspring are weaned, toe clipping for identification and tail snipping for genomic DNA extraction are performed. Mice identified as homozygotes are cohoused for expansion breeding. The mice used in this article are littermate homozygotes (6-8 weeks).<br>Other C57BL/6J mice (6-8 weeks) were purchased from Shanghai Model Organisms. All mice were reared in-house (temperature:

	20-25 °C, humidity: 40-60%) in a 12-h light-dark cycle under specific pathogen-free conditions. All animals had free access to food and sterilized water.<br>In the study of nascent hepatic proteomics under physiological conditions, both female and male mice aged 8 to 10 weeks were included to more accurately reflect the physiological state. For the investigation of alcohol-induced liver injury, male mice aged 8 to 10 weeks were selected to eliminate the potential influence of female hormonal fluctuations on experimental outcomes, which was also based on established protocols used in standard models of alcoholic liver injury include Lieber-DeCarli liquid diet model.
Wild animals	This study did not involve wild animals.
Reporting on sex	In vivo experiments in mice were performed in male mice to exclude sex-bias effect in this study.
Field-collected samples	This study did not involve field-collected samples.
Ethics oversight	The care of the experimental animals was in accordance with the guidelines of, and approved by, the Institutional Animal Care and Use Committee of Zhejiang University.

Note that full information on the approval of the study protocol must also be provided in the manuscript.

Plants

Seed stocks	N/A
Novel plant genotypes	N/A
Authentication	N/A

Flow Cytometry

Plots

Confirm that:

- [x] The axis labels state the marker and fluorochrome used (e.g. CD4-FITC).
- [x] The axis scales are clearly visible. Include numbers along axes only for bottom left plot of group (a 'group' is an analysis of identical markers).
- [x] All plots are contour plots with outliers or pseudocolor plots.
- [x] A numerical value for number of cells or percentage (with statistics) is provided.

Methodology

Sample preparation	HEK293T were seeded in a 12-well plate and grown to 50-60% confluence for transfection. Cells were co-transfected with the pCMV vector and the pEGFP-mCherry-T2A-EGFP-190TAG at a ratio of 1:1 (µg:µg). Transfections were performed by lip2000 reagent (BioSharp) according to the manufacturer’s protocol with or without the addition of the corresponding amino acids. At 48 h post-transfection, cells were trypsinized and neutralized by the complete medium before centrifugation. Cells were centrifuged at 200 ×g for 3 min, washed and resuspended in PBS for FACS analysis.<br><br>HepG2 cells and AML12 cells were seeded in a 6-well plate and reached 50-60% confluency. HepG2 cells were treated with 8 µM Triacsin C (Aladdin) for 3 h and 3% ethanol for the next 3 h sealed with parafilm. AML12 cells were treated with 10µM Tracsin C for 24h and 1% ethanol for next 24h sealed with parafilm. To quantify the lipid droplets, Nile Red solution (MedChem Express) was added to the cells, and then incubated at room temperature for 30 min. The cells were washed and resuspended with PBS.
Instrument	Beckman CytoFlex
Software	Collected with CytExpert (version 2.0.0.153, Beckman Coulter) and analyzed by FlowJo V10 (version 14.0.0.0, Flexera). Software)
Cell population abundance	At least 50,000 single cells were analyzed per condition for amber suppression efficiency assessment, and at least 10,000 single cells were analyzed per condition for Nile Red staining. Purity of HEK293T, HepG2 and AML12 cells is 99% or higher.

Gating strategy

HEK293T, HepG2 and AML12 cells were used to set appropriate FSC and SSC gains. The fluorescent protein expressed cells were used to set FITC and PE gains and gate. Nile Red staining in parental HepG2 and AML12 cells was used to set ECD-A gains and gate.

[x] Tick this box to confirm that a figure exemplifying the gating strategy is provided in the Supplementary Information.
